# Integrated transcriptome, small RNA, and degradome analysis reveals the complex network regulating starch biosynthesis in maize

**DOI:** 10.1186/s12864-019-5945-1

**Published:** 2019-07-11

**Authors:** Xiaocong Zhang, Sidi Xie, Jienan Han, Yu Zhou, Chang Liu, Zhiqiang Zhou, Feifei Wang, Zixiang Cheng, Junjie Zhang, Yufeng Hu, Zhuanfang Hao, Mingshun Li, Degui Zhang, Hongjun Yong, Yubi Huang, Jianfeng Weng, Xinhai Li

**Affiliations:** 1 Institute of Crop Science, Chinese Academy of Agricultural Sciences, Beijing, China; 2 College of Agronomy, Sichuan Agricultural University, Chengdu, Sichuan China; 3 College of Agronomy, Northeast Agricultural University, Harbin, Heilongjiang China; 4 College of Life Science, Sichuan Agricultural University, Ya’an, Sichuan China

**Keywords:** Maize, Endosperm, Starch biosynthesis, miRNA, Regulatory network

## Abstract

**Background:**

Starch biosynthesis in endosperm is a key process influencing grain yield and quality in maize. Although a number of starch biosynthetic genes have been well characterized, the mechanisms by which the expression of these genes is regulated, especially in regard to microRNAs (miRNAs), remain largely unclear.

**Results:**

Sequence data for small RNAs, degradome, and transcriptome of maize endosperm at 15 and 25 d after pollination (DAP) from inbred lines Mo17 and Ji419, which exhibit distinct starch content and starch granule structure, revealed the mediation of starch biosynthetic pathways by miRNAs. Transcriptome analysis of these two lines indicated that 33 of 40 starch biosynthetic genes were differentially expressed, of which 12 were up-regulated in Ji419 at 15 DAP, one was up-regulated in Ji419 at 25 DAP, 14 were up-regulated in Ji419 at both 15 and 25 DAP, one was down-regulated in Ji419 at 15 DAP, two were down-regulated in Ji419 at 25 DAP, and three were up-regulated in Ji419 at 15 DAP and down-regulated in Ji419 at 25 DAP, compared with Mo17. Through combined analyses of small RNA and degradome sequences, 22 differentially expressed miRNAs were identified, including 14 known and eight previously unknown miRNAs that could target 35 genes. Furthermore, a complex co-expression regulatory network was constructed, in which 19 miRNAs could modulate starch biosynthesis in endosperm by tuning the expression of 19 target genes. Moreover, the potential operation of four miRNA-mediated pathways involving transcription factors, miR169a-*NF-YA1*-*GBSSI*/*SSIIIa* and miR169o-*GATA9*-*SSIIIa*/*SBEIIb*, was validated via analyses of expression pattern, transient transformation assays, and transactivation assays.

**Conclusion:**

Our results suggest that miRNAs play a critical role in starch biosynthesis in endosperm, and that miRNA-mediated networks could modulate starch biosynthesis in this tissue. These results have provided important insights into the molecular mechanism of starch biosynthesis in developing maize endosperm.

**Electronic supplementary material:**

The online version of this article (10.1186/s12864-019-5945-1) contains supplementary material, which is available to authorized users.

## Background

Maize (*Zea mays* L.) is grown extensively as a staple food and feed crop around the world. Starch is the most important component of maize endosperm, accounting for 70% of dry endosperm weight. The accumulation of starch in endosperm is one of the major factors determining maize yield, and the physical and chemical properties of starch can influence maize quality [1].

Starch biosynthesis is catalyzed by multiple, well-characterized enzymes, including ADP-glucose pyrophosphorylase (AGP), granule-bound starch synthase (GBSS), soluble starch synthase (SS), starch branching enzyme (SBE), starch debranching enzyme (DBE), and starch phosphorylase (PHO) [2–4]. Thus far, many genes associated with endosperm development have been cloned in maize. Among these genes, *shrunken2* (*Sh2*), *brittle2* (*Bt2*), *GBSSI*, *SSI*, *SSIIa*, *SSIIIa*, *SBEI*, *SBEIIa*, *SBEIIb*, and *isoamylase 1* (*ISA1*) are predominantly involved in starch biosynthesis [2, 5–7]. Although the functions of many genes involved in starch biosynthesis are well understood, knowledge of the regulation of the expression of these genes remains limited.

As one of the main regulators of gene expression, transcription factors activate or repress the transcription of downstream genes upon binding to *cis*-acting elements in promoter regions. Only a few studies of the direct regulation of starch biosynthesis by transcription factors in cereals have been published. In barley, a WRKY transcription factor SUSIBA2 (sugar signaling in barley) can bind to the promoter of *ISA1* to regulate its expression [8]. In maize, several transcription factors regulate the expression of starch biosynthetic genes. For example, ZmaNAC36 can regulate the expression of *AGP large subunit 2* (*AGPL2*), *AGP small subunit 2* (*AGPS2*), *SSI*, *GBSSIIb*, and *SBEI* [9]. ZmbZIP91 can regulate the expression of *Bt2*, *SSI*, *SSIIIa*, and *ISA1* [10]; and ZmEREB156 can regulate the expression of *Sh2* and *SSIIIa* [11]. Further, *opaque2* (*O2*) and *prolamine-box binding factor* (*PBF*) can regulate the expression of *pyruvate orthophosphate dikinase 1* (*PPDK1*), *PPDK2*, and *SSIII* [12].

Many metabolic processes in plants can be regulated by phytohormones [13]. Auxin (IAA), abscisic acid (ABA), brassinosteroid (BR), and gibberellin (GA) play important roles in starch biosynthesis and grain development. Palacios et al. [14] found that the AGP activity and starch content in *Chlorella sorokiniana* strains with normal levels of IAA accumulation were higher than in those with abnormal levels of IAA accumulation. In *Arabidopsis*, the sucrose induced expression of *AGPL3* and *SBE2.2* could be enhanced by ABA [15]. Similarly, the expression of *SSI* could be induced by ABA in maize endosperm [16]. In rice, increased BR increases UDP-glucose pyrophosphorylase (UGP) activity and starch content in grain [17]. During rice grain filling, higher GA content can lead to lower activities of AGP and other starch biosynthetic enzymes in grain and inhibit the accumulation of starch [18].

MicroRNAs (miRNAs) are a class of short non-coding RNAs that can modulate gene expression post-transcriptionally through sequence complementarity [19]. At present, there are only a few reports that miRNAs participate in the regulation of starch biosynthesis. miR394 can target sugar metabolism-related genes to regulate starch biosynthesis in cassava [20]. Overexpression of miR156 has increased starch content in plants such as *Arabidopsis*, maize, and switchgrass [21]. Recently, miRNAs have been shown to play an important role in grain development and grain filling. In rice, miR167 can regulate grain development through the auxin-miR167-ARF8 (auxin response factor)-OsGH3.2 (GH3, an auxin-binding protein) pathway [22]. Moreover, this pathway, together with the miR160-, miR164-, and miR390-mediated auxin signaling pathways, was confirmed to regulate grain filling in rice [23]. In addition, the miRNAs identified during endosperm development [24–26], grain filling [27], and kernel development [28] could have roles in starch accumulation in maize. miRNAs might modulate starch biosynthesis, which is critical for endosperm and grain development, by tuning the expression of transcription factors or mediating the effects of plant hormones.

Co-expression analyses have shown that co-expressed genes, those with similar expression profiles, can be involved in similar biological functions, which provides a feasible means to discover new genes [29, 30]. A recent study has reported a newly discovered starch synthase gene *SSV* by co-expression analysis [31]. Co-expression analysis is also an effective approach to identify genes related to a specific metabolic pathway. For instance, *Rice Starch Regulator 1* (*RSR1*) in rice [32], and *NAC36* and *bZIP91* in maize [9, 10] were identified to regulate starch biosynthesis by co-expression analysis. Additionally, gene co-expression network analysis can be conducted using high-throughput gene expression profile data. Using available RNA sequencing (RNA-Seq) data, Huang et al. [33] constructed and optimized a large scale gene co-expression network in maize, and predicted a cell wall biosynthesis pathway from the merged network. Co-expression analysis has great utility for gene identification and functional analysis, and is an important strategy for enhancing our understanding of regulatory pathways.

There are few reports of the use of integrated high-throughput sequence data to unravel miRNA-mediated regulatory networks in developing maize endosperm. The present study analyzed small RNA, degradome, and transcriptome sequence data for endosperm at 15 and 25 d after pollination (DAP) from the maize inbred line Mo17 and its improved line Ji419, which exhibit distinct endosperm starch content and starch granule structure. These analyses identified and characterized differentially expressed miRNAs and their targets, and further, allowed construction of a co-expression regulatory network for starch biosynthesis in maize endosperm. Moreover, four miRNA-mediated regulatory pathways were chosen to implement expression pattern analysis, transient overexpression analysis, and transactivation assays to validate the modulating network. Our results will provide valuable information for a better understanding of the molecular mechanism of starch biosynthesis in maize endosperm.

## Results

### Transcriptome analysis of developing maize endosperm

Transcriptome analysis of developing endosperm revealed a total of 9241 differentially expressed genes (DEGs) between Mo17 and Ji419 at 15 DAP, of which 4590 genes were up-regulated and 4651 genes were down-regulated in Ji419 compared with Mo17. At 25 DAP, 1149 genes were differentially expressed, of which 552 genes were up-regulated and 597 genes were down-regulated in Ji419 compared with Mo17.

Gene Ontology (GO) term enrichment analysis suggested that the DEGs between Mo17 and Ji419 at 15 DAP were significantly enriched in biological processes including ‘glycogen biosynthesis’, cellular components such as ‘chloroplast’, and molecular functions including ‘catalytic activity’ (Additional file 1: Figure S1a, b). At 25 DAP, the DEGs were significantly enriched in biological processes including ‘auxin transport’, cellular components such as ‘phosphopyruvate hydratase’, and molecular functions including ‘phosphoenolpyruvate carboxylase activity’.

Additionally, Kyoto Encyclopedia of Genes and Genomes (KEGG) pathway enrichment analysis showed that the DEGs between Mo17 and Ji419 at 15 DAP were significantly enriched in biological pathways including ‘carbon metabolism’, ‘pyruvate metabolism’, and ‘glycolysis/gluconeogenesis’ (Additional file 1: Figure S1c, d). At 25 DAP, the DEGs were significantly enriched in biological pathways such as ‘carbon fixation in photosynthetic organisms’, ‘pyruvate metabolism’, ‘brassinosteroid biosynthesis’, and ‘other glycan degradation’.

### Expression profiles of starch biosynthetic genes in developing endosperm

**Fig. 1:**
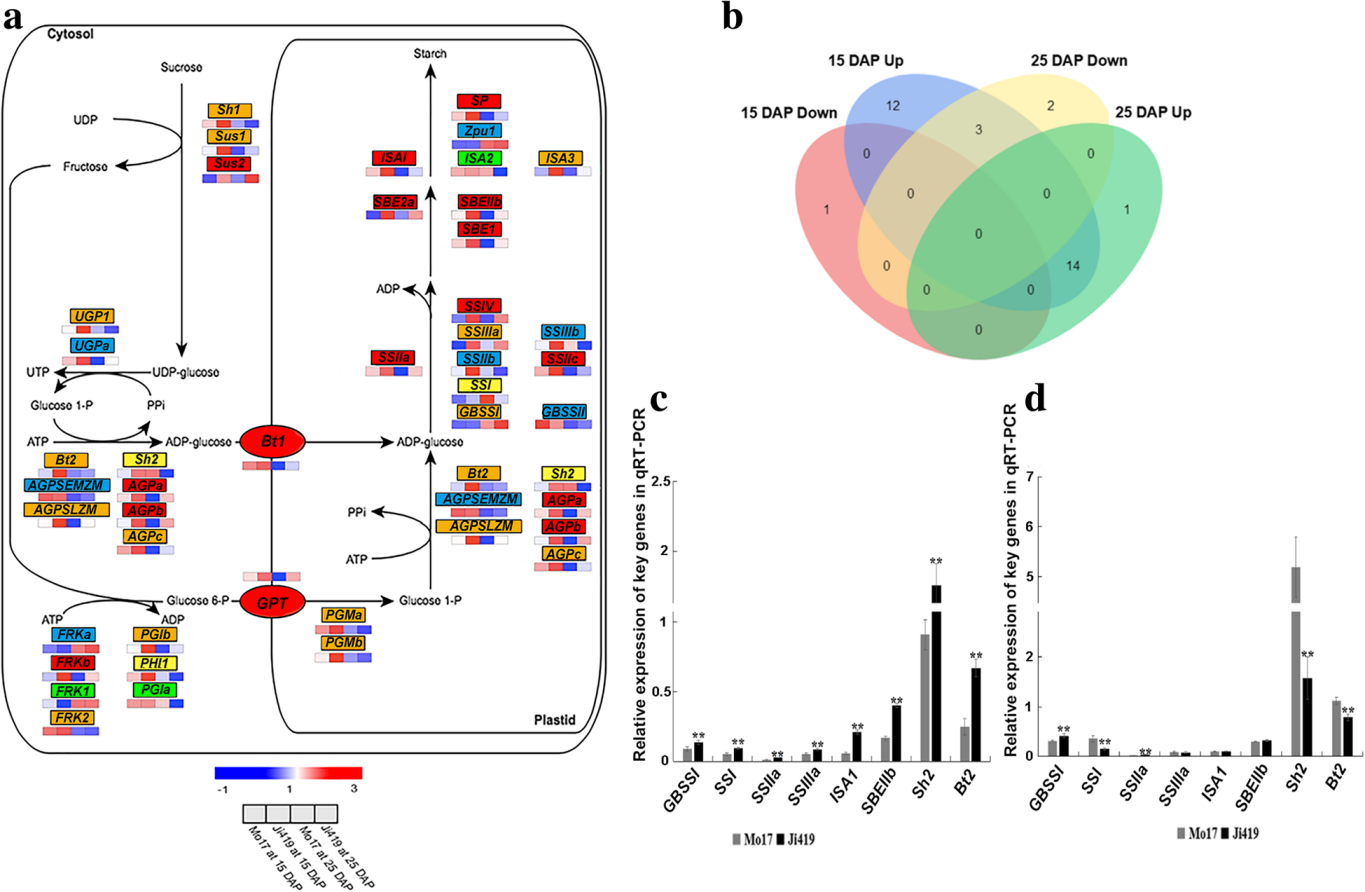
Expression profiles of starch biosynthetic genes. **a** The expression of 40 known starch biosynthetic genes and the pathways in which they could be involved in the endosperm of maize inbred lines Mo17 and Ji419 at 15 and 25 DAP. The heat map represents the expression of each gene in a specific line. Red boxes represent up-regulated genes in Ji419 compared with Mo17 at both 15 and 25 DAP; orange boxes represent genes up-regulated in Ji419 at only one stage; yellow boxes represent genes up-regulated in Ji419 at one stage, but down-regulated at the other stage; green boxes represent genes down-regulated in Ji419 at only one stage; blue boxes represent genes with no differential expression between Mo17 and Ji419 at both 15 and 25 DAP. **b** Venn diagram of starch biosynthetic genes that are differentially expressed in the endosperm of Mo17 and Ji419 at 15 and 25 DAP. Red represents genes down-regulated in Ji419 compared with Mo17 at 15 DAP; blue represents genes up-regulated in Ji419 at 15 DAP; yellow represents genes down-regulated in Ji419 at 25 DAP; green represents genes up-regulated in Ji419 at 25 DAP. **c-d** Expression levels of eight key starch biosynthetic genes in the endosperm of Mo17 and Ji419 at 15 DAP (**c**) and 25 DAP (**d**). Black bars represent the expression of genes in Ji419; grey bars represent the expression of genes in Mo17. The data in **c** and **d** are means ± SD (*n* = 3). ** significant at *p* ≤ 0.01 by the Student’s *t* test

RNA-Seq analysis showed that 40 known starch biosynthetic genes were enriched in functional categories including ‘starch biosynthesis’, ‘glycogen biosynthesis’, ‘carbon metabolism’, and ‘glycolysis/gluconeogenesis’ (Additional file 2: Tables S1 and S2). The functions of these 40 genes, and the starch biosynthetic pathways in which they could be involved, are shown in Fig. 1a.

Among these 40 genes, 33 genes were differentially expressed, 12 genes were up-regulated only in Ji419 at 15 DAP, one gene was up-regulated only in Ji419 at 25 DAP, one gene was down-regulated only in Ji419 at 15 DAP, and two genes were down-regulated only in Ji419 at 25 DAP, compared to Mo17 (Fig. 1b). At both 15 and 25 DAP, 14 genes, including six starch biosynthetic genes *SSIIa*, *SSIV*, *ISA1*, *SBEI*, *SBEIIa*, and *SBEIIb*, were up-regulated in Ji419. In addition, three genes including *Sh2*, *SSI*, and *phosphohexose isomerase1* (*PHI1*) were up-regulated in Ji419 at 15 DAP and down-regulated in Ji419 at 25 DAP (Fig. 1a, b).

Quantitative real-time polymerase chain reaction (qRT-PCR) analysis showed that in Ji419 at 15 DAP, all eight key starch biosynthetic genes (*GBSSI*, *SSI*, *SSIIa*, *SSIIIa*, *ISA1*, *SBEIIb*, *Sh2*, and *Bt2*) were significantly up-regulated, and that in Ji419 at 25 DAP, *GBSSI* and *SSIIa* were significantly up-regulated, while *SSI*, *Sh2* and *Bt2* were significantly down-regulated compared with Mo17 (Fig. 1c, d), concordantly with the expression profiles obtained from RNA-Seq data (Additional file 2: Table S1).

**Fig. 2:**
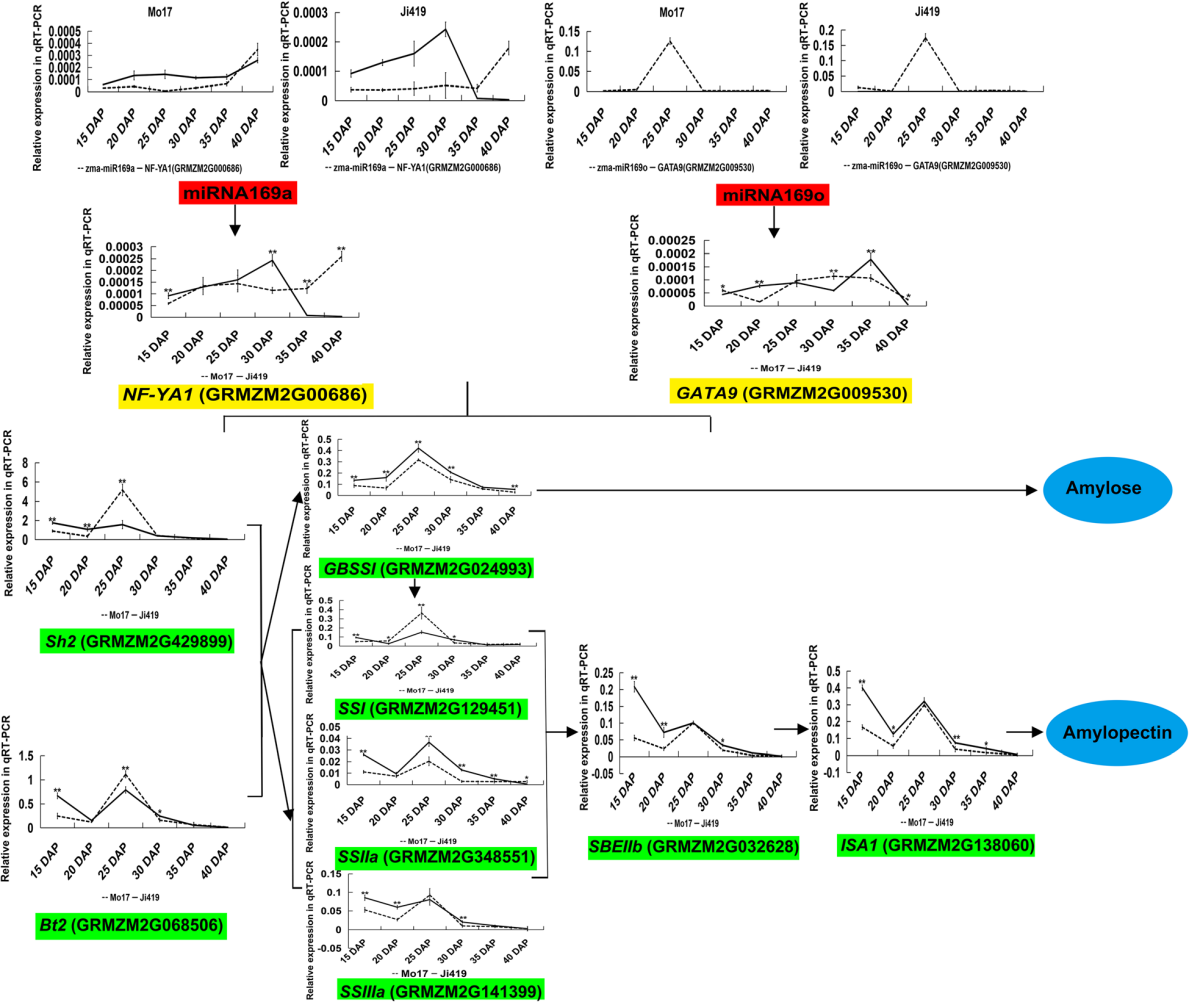
Analysis of the expression patterns of two miRNAs and their target genes, and eight key starch biosynthetic genes in the endosperm of maize inbred lines Mo17 and Ji419 at 15, 20, 25, 30, 35, and 40 DAP. Red boxes represent miRNAs, yellow boxes represent target genes, and green boxes represent starch biosynthetic genes

Additionally, similar expression patterns were observed in these eight genes at six endosperm developmental stages: the transcript levels of these genes decreased from 15 to 20 DAP, increased from 20 to 25 DAP, and continuously decreased from 25 to 40 DAP (Fig. 2). The expression peak for the majority of these genes occurred at 15 or 25 DAP, indicating that starch metabolism in the endosperm of Ji419 and Mo17 might be more dynamic at these two stages. These eight genes could be further classified into two groups based on their expression patterns. One group includes *SSI*, *SSIIIa*, *Sh2*, and *Bt2*, which showed similar or higher expression in Ji419 compared with Mo17 at five developmental stages excluding 25 DAP. The other group includes *GBSSI*, *SSIIa*, *ISA1*, and *SBEIIb*, which exhibited higher expression in Ji419 than in Mo17 in all six developmental stages, which was consistent with the significantly higher starch content in the mature endosperm of Ji419 than in that of Mo17 (Additional file 3: Figure S2).

### Differential expression of miRNAs in developing endosperm

Sequencing of small RNAs generated 16,968,986 raw reads representing 5,065,474 unique reads from the endosperm of Mo17 at 15 DAP, and 14,965,571 raw reads representing 2,470,691 unique reads from the endosperm of Ji419. A further 15,215,476 raw reads representing 3,885,746 unique reads were generated from Mo17 endosperm at 25 DAP, and 16,315,425 raw reads representing 414,202 unique reads were generated from Ji419 endosperm at 25 DAP. In total, 314 miRNAs were detected, including 180 known miRNAs and 134 previously unknown ones. Most of these miRNAs ranged from 20 to 24 nt in length (Additional file 4: Figure S3a).

A total of 129 differentially expressed miRNAs were identified between Mo17 and Ji419 at 15 DAP, including 52 known miRNAs belonging to 21 miRNA families, and 77 newly discovered miRNAs (Additional file 2: Table S3). Among the 52 known miRNAs, 38 were down-regulated in Ji419 compared with Mo17, 22 of which were down-regulated by at least 3-fold. For example, miR528a-3p_L + 1 was down-regulated by 20-fold. Another 14 miRNAs were up-regulated in Ji419, of which seven were up-regulated by at least 3-fold, such as miR319b-5p, which was up-regulated by 17-fold. Among the 77 previously unknown miRNAs, 64 were down-regulated in Ji419, 31 of these were down-regulated by at least 3-fold, and PC-3p-104553_20 was down-regulated by 20-fold. Another 13 miRNAs were up-regulated in Ji419, while PC-3p-1338_720 was up-regulated by 253-fold.

A total of 131 differentially expressed miRNAs were identified between the two inbred lines at 25 DAP, including 52 known miRNAs belonging to 20 miRNA families and 79 newly discovered miRNAs (Additional file 2: Table S4). Among the 52 known miRNAs, 38 were down-regulated in Ji419 compared with Mo17, 29 of which were down-regulated by at least 3-fold. Among these, miR528a-3p_L + 1 and miR2118b were both down-regulated by 10-fold. In addition, 14 other miRNAs were up-regulated in Ji419, 10 of which were up-regulated by at least 3-fold, and one, miR159c-5p_L-1R-1, was up-regulated by 33-fold. Among the 79 newly discovered miRNAs, 68 were down-regulated in Ji419, 60 of which were down-regulated by at least 3-fold. Among these, PC-3p-383_2667, PC-5p-383_2667, and PC-5p-28889_59 were down-regulated by 10-fold. A further 11 miRNAs were up-regulated in Ji419, with PC-3p-1338_720 up-regulated by 170-fold.

Among these differentially expressed miRNAs, 35 and 11 were down- and up-regulated only in Ji419 at 15 DAP, respectively, while 39 and nine were down- and up-regulated only in Ji419 at 25 DAP, respectively (Additional file 4: Figure S3b). Meanwhile, 64 miRNAs were down-regulated and 13 were up-regulated in Ji419 at both 15 and 25 DAP. In addition, three miRNAs were up-regulated in Ji419 at 15 DAP and down-regulated in Ji419 at 25 DAP, while another three were down-regulated in Ji419 at 15 DAP and up-regulated in Ji419 at 25 DAP.

### Targets of differentially expressed miRNAs in developing endosperm

Through degradome sequencing, 2.0 × 10^7^ raw reads representing 2.9 × 10^6^ unique reads were generated from the mixed degradome pools. In total, 55 target genes with 97 transcripts modulated by 56 miRNAs were identified.

**Fig. 3:**
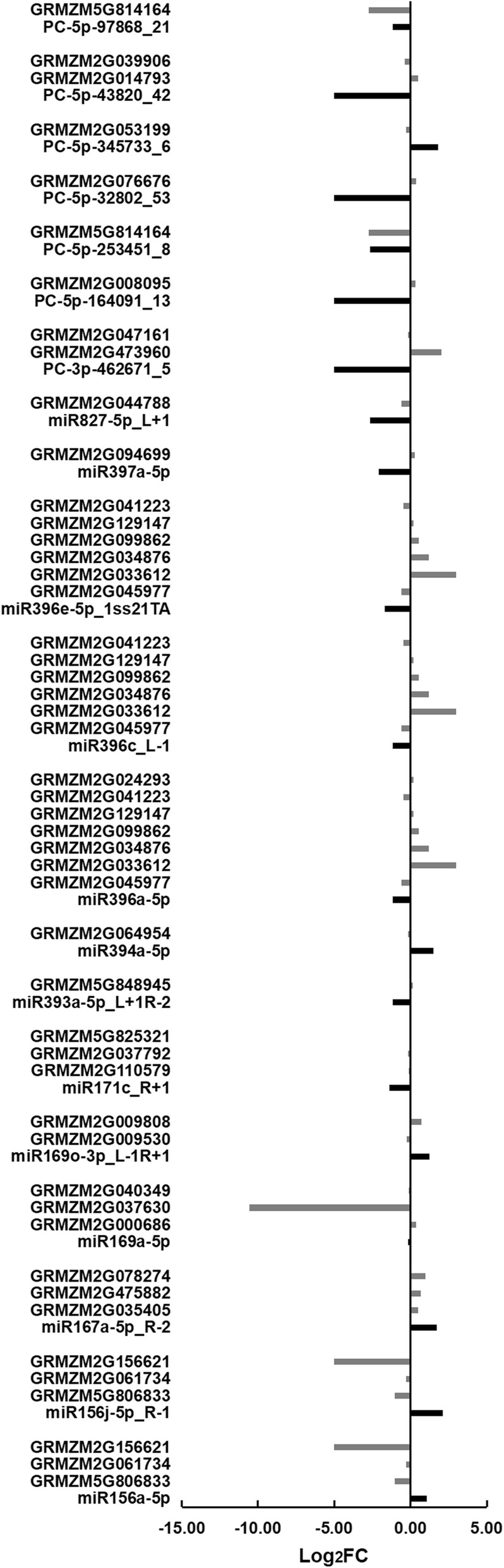
Differential expression of miRNAs and their target genes in the endosperm of maize inbred lines Mo17 and Ji419 at 15 DAP. FC, fold-change of the expression of the miRNAs/target genes in Ji419 relative to that in Mo17 at 15 DAP based on the sequencing data. Black bars represent expression of miRNAs; grey bars represent expression of target genes

**Fig. 4:**
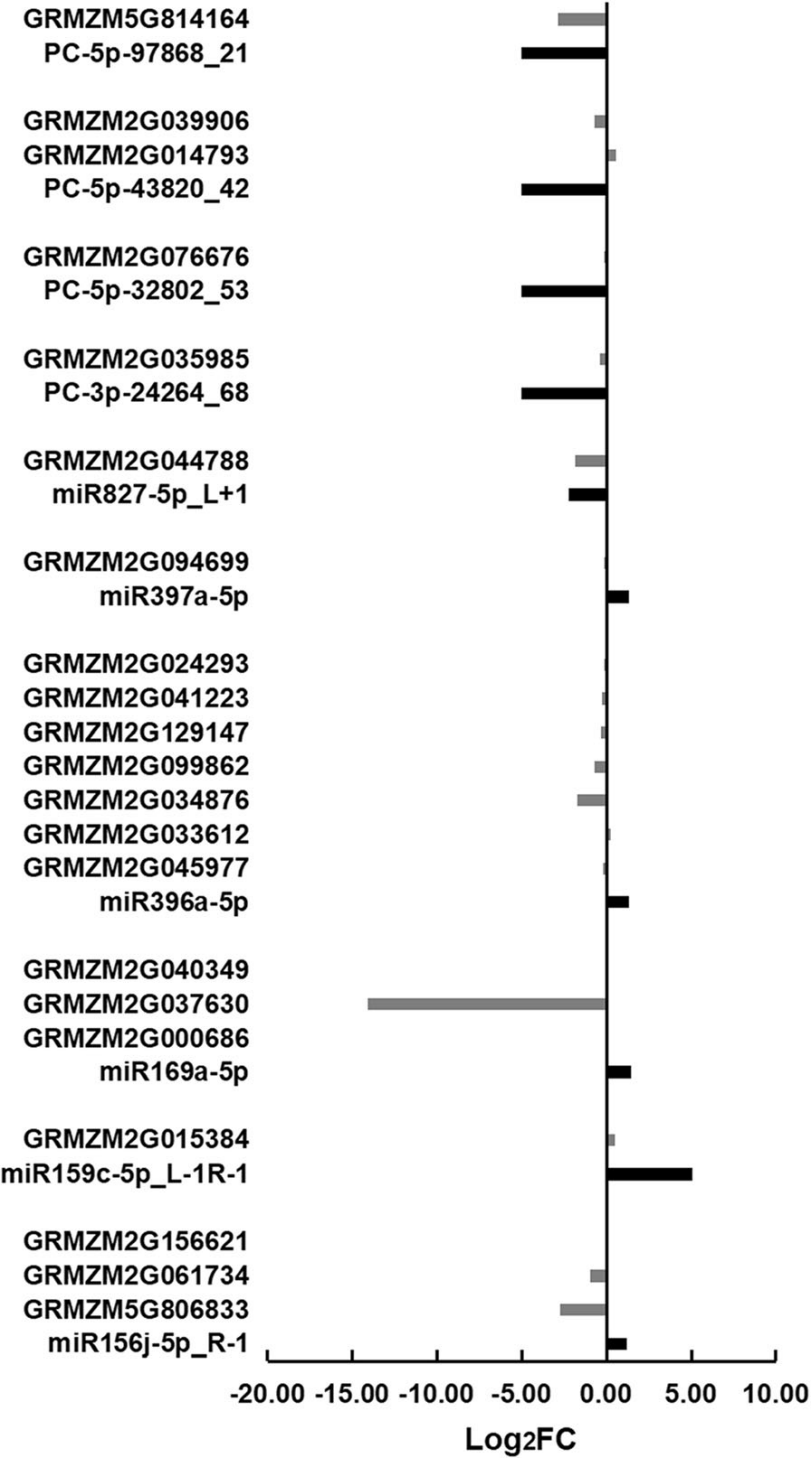
Differential expression of miRNAs and their target genes in the endosperm of maize inbred lines Mo17 and Ji419 at 25 DAP. FC, fold-change of the expression of the miRNAs/target genes in Ji419 relative to that in Mo17 at 25 DAP based on the sequencing data. Black bars represent expression of miRNAs; grey bars represent expression of target genes

Target genes of these differentially expressed miRNAs were further screened. As a result, 26 target genes with 47 transcripts regulated by 14 known miRNAs were detected (Figs. 3 and 4; Additional file 2: Tables S5 and S6). Of these miRNAs, both miR156a-5p and miR156j-5p_R-1 could target GRMZM5G806833 (unknown function), GRMZM2G061734 (*squamosa promoter-binding protein transcription factor 5*, *SBP5*), and GRMZM2G156621 (*SBP31*); miR159c-5p_L-1R-1 could target GRMZM2G015384 (*actin related protein 4*); miR167a-5p_R-2 could target GRMZM2G035405 (*ARF18*), GRMZM2G475882 (*ARF30*), and GRMZM2G078274 (*ARF6*); miR169a-5p could target GRMZM2G000686 (*nuclear transcription factor Y subunit A1*, *NF-YA1*), GRMZM2G037630 (*CCAAT-HAP2-transcription factor 23*, *CA2P3*), and GRMZM2G040349 (*CCAAT-HAP2-transcription factor 24*, *CA2P4*); miR169o-3p_L-1R + 1 could target GRMZM2G009530 (*C2C2-GATA-transcription factor 9*, *GATA9*) and GRMZM2G009808 (*aconitase3*); miR171c_R + 1 could target three GRAS (GIBBERELLIN-ACID INSENSITIVE (GAI), REPRESSOR OF GAI (RGA), SCARECROW (SCR)) family transcription factors (GRMZM2G110579, GRMZM5G825321, and GRMZM2G037792); miR393a-5p_L + 1R-2 could target GRMZM5G848945 (*auxin signaling F-box*, *AFB*); miR394a-5p could target GRMZM2G064954 (*F-box protein 6*); and miR396a-5p, miR396c_L-1, or miR396e-5p_1ss21TA could target six growth-regulating factors (GRMZM2G045977, GRMZM2G033612, GRMZM2G034876, GRMZM2G099862, GRMZM2G129147, and GRMZM2G041223). Also, miR396a-5p could regulate GRMZM2G024293 (encoding a nucleoside triphosphate hydrolase). In addition, miR397a-5p and miR827-5p_L + 1 could modulate GRMZM2G094699 (*coatomer protein complex subunit beta 1*, *COPB1*) and GRMZM2G044788 (unknown function), respectively.

Nine target genes with 47 transcripts were detected, which were regulated by eight newly discovered miRNAs including PC-3p-24264_68 and PC-5p-164091_13 (Figs. 3 and 4; Additional file 2: Tables S5 and S6). Of these miRNAs, both PC-5p-32802_53 and PC-5p-43820_42 were specifically expressed in Mo17 at 15 and 25 DAP, with PC-5p-32802_53 targeting GRMZM2G076676 (encoding a glycine-rich protein), and PC-5p-43820_42 targeting both GRMZM2G014793 (encoding a glycosyl transferase) and GRMZM2G039906 (encoding a cell cycle control protein). Both PC-3p-462671_5 and PC-5p-164091_13 were specifically expressed in Mo17 at 15 DAP, with PC-3p-462671_5 targeting both GRMZM2G473960 (encoding a seed maturation protein) and GRMZM2G047161 (unknown function), and PC-5p-164091_13 targeting GRMZM2G008095 (encoding a vesicle transport protein). Both PC-5p-97868_21 and PC-3p-24264_68 were specifically expressed in Mo17 at 25 DAP, with PC-5p-97868_21 targeting GRMZM5G814164 (*peroxin3*), and PC-3p-24264_68 targeting GRMZM2G035985 (encoding a transduction protein). Additionally, PC-5p-253451_8 and PC-5p-345733_6 could regulate GRMZM5G814164 and GRMZM2G053199 (encoding a protein phosphatase), respectively. Notably, GRMZM5G814164 could be regulated by both PC-5p-97868_21 and PC-5p-253451_8.

Among all the miRNA-target pairs, 29 pairs including miR393a-5p_L + 1R-2-GRMZM5G848945, miR169o-3p_L-1R + 1-GRMZM2G009530, and miR169a-5p-GRMZM2G000686, and eight pairs including PC-5p-43820_42-GRMZM2G014793, miR396a-5p-GRMZM2G034876, and miR169a-5p-GRMZM2G037630, showed reverse expression patterns at 15 and 25 DAP, respectively (Figs. 3 and 4).

None of the starch biosynthetic genes that were directly regulated by miRNAs were detected through degradome sequencing. However, bioinformatic prediction showed that the starch synthase genes *SSIIIa* and *SSIIb-2* might be the targets of two newly discovered miRNAs, PC-3P-336668_6 and PC-3p-169926_13, respectively (Additional file 2: Table S7).

### Co-expression network of genes involved in starch biosynthesis in developing endosperm

**Fig. 5:**
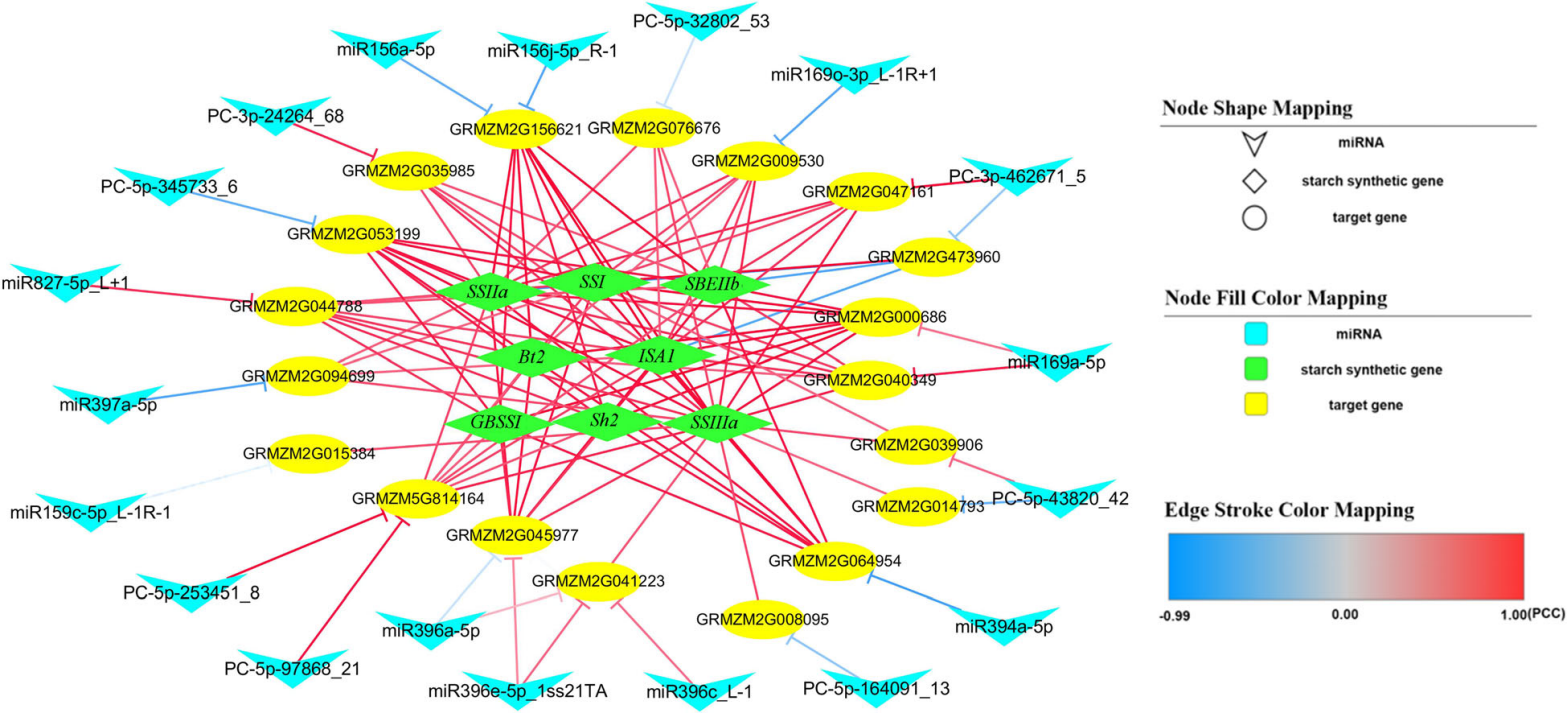
Co-expression network of miRNA-target-starch biosynthesis in developing maize endosperm. Blue boxes represent miRNAs, yellow boxes represent target genes, and green boxes represent starch biosynthetic genes. PCC, Pearson’s correlation coefficient

A set of 35 target genes was identified via combined analysis of the degradome and differentially expressed miRNAs. Co-expression analysis of these 35 genes and eight key genes involved in maize endosperm starch biosynthesis suggested that 34 target genes were significantly co-expressed with at least one of the eight starch biosynthetic genes (Additional file 5: Figure S4; Additional file 2: Table S8). Co-expression analysis revealed a complex network modulating starch biosynthesis in developing maize endosperm, in which 19 miRNAs, including 11 known and eight newly discovered miRNAs, could regulate 19 target genes associated with starch biosynthesis (Fig. 5). For example, miR169a and miR169o could directly target *NF-YA1* and *GATA9*, respectively, to control starch biosynthetic genes.

### Validation of the regulatory pathways for starch biosynthesis mediated by miR169a-*NF-YA1* and miR169o-*GATA9*

To verify the regulatory pathways involved in starch biosynthesis, the expression patterns of the miRNA-target pairs miR169a-*NF-YA1* and miR169o-*GATA9* were analyzed at six endosperm developmental stages by qRT-PCR (Fig. 2). The results revealed a reverse expression pattern for miR169a-*NF-YA1* during endosperm development. The expression of miR169o was relatively high, whereas that of *GATA9* was extremely low, implying that miR169o might repress the expression of *GATA9*.

**Fig. 6:**
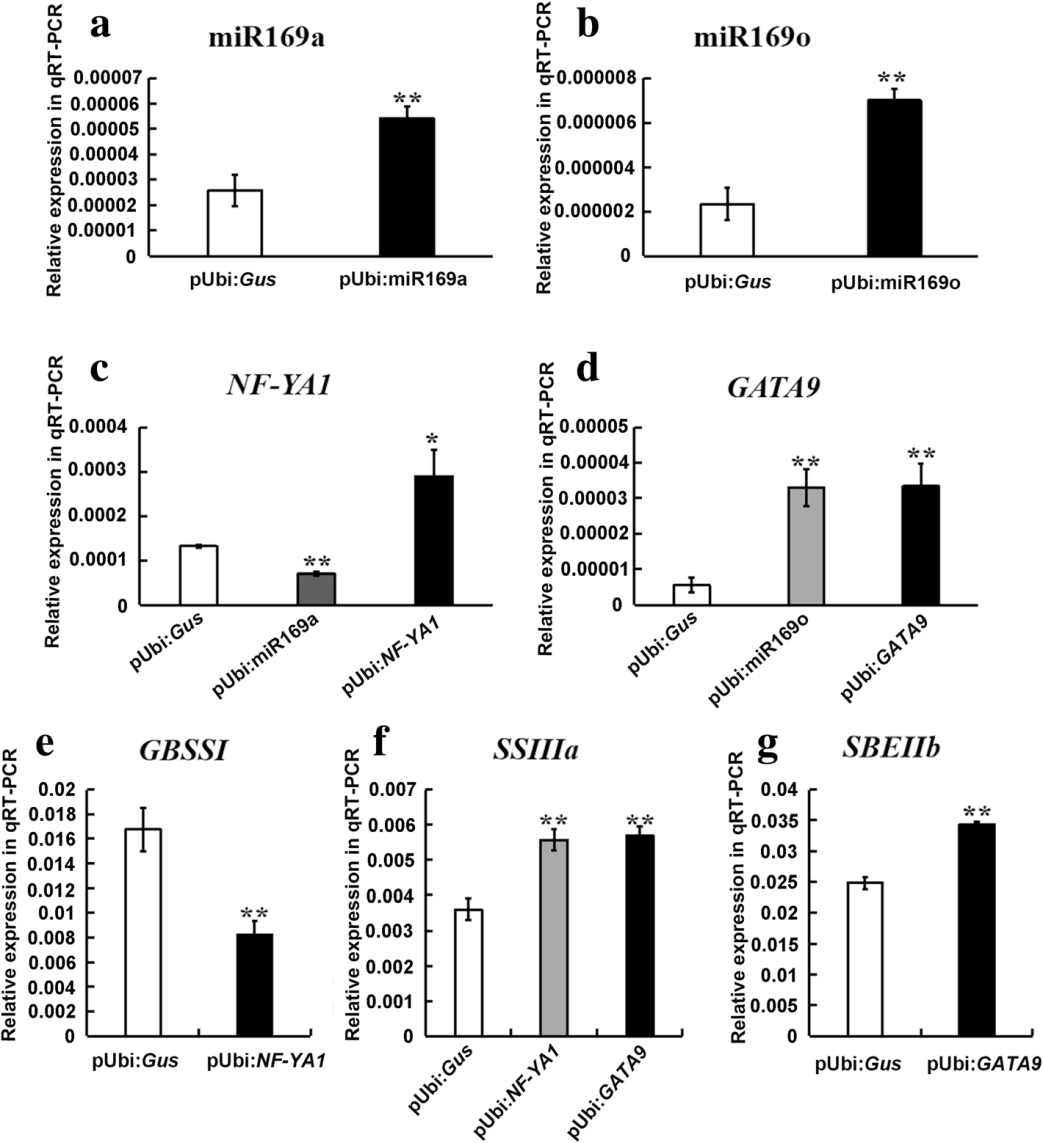
Transient expression analysis in maize endosperm. **a** The expression of miR169a in miR169a-overexpressing endosperm (pUbi:miR169a) and the control (pUbi:*Gus*); **b** The expression of miR169o in miR169o-overexpressing endosperm (pUbi:miR169o) and the control (pUbi:*Gus*); **c** The expression of *NF-YA1* in miR169a/*NF-YA1*-overexpressing endosperm (pUbi:miR169a and pUbi:*NF-YA1*) and the control (pUbi:*Gus*); **d** The expression of *GATA9* in miR169o/*GATA9*-overexpressing endosperm (pUbi:miR169o and pUbi:*GATA9*) and the control (pUbi:*Gus*); **e** The expression of *GBSSI* in *NF-YA1*-overexpressing endosperm (pUbi:*NF-YA1*) and the control (pUbi:*Gus*); **f** The expression of *SSIIIa* in *NF-YA1*/*GATA9*-overexpressing endosperm (pUbi:*NF-YA1* and pUbi:*GATA9*) and the control (pUbi:*Gus*); **g** The expression of *SBEIIb* in *GATA9*-overexpressing endosperm (pUbi:*GATA9*) and the control (pUbi:*Gus*). All data are means ± SD (*n* = 3). *, ** significant at *p* ≤ 0.05 and *p* ≤ 0.01 by the Student’s *t* test

**Fig. 7:**
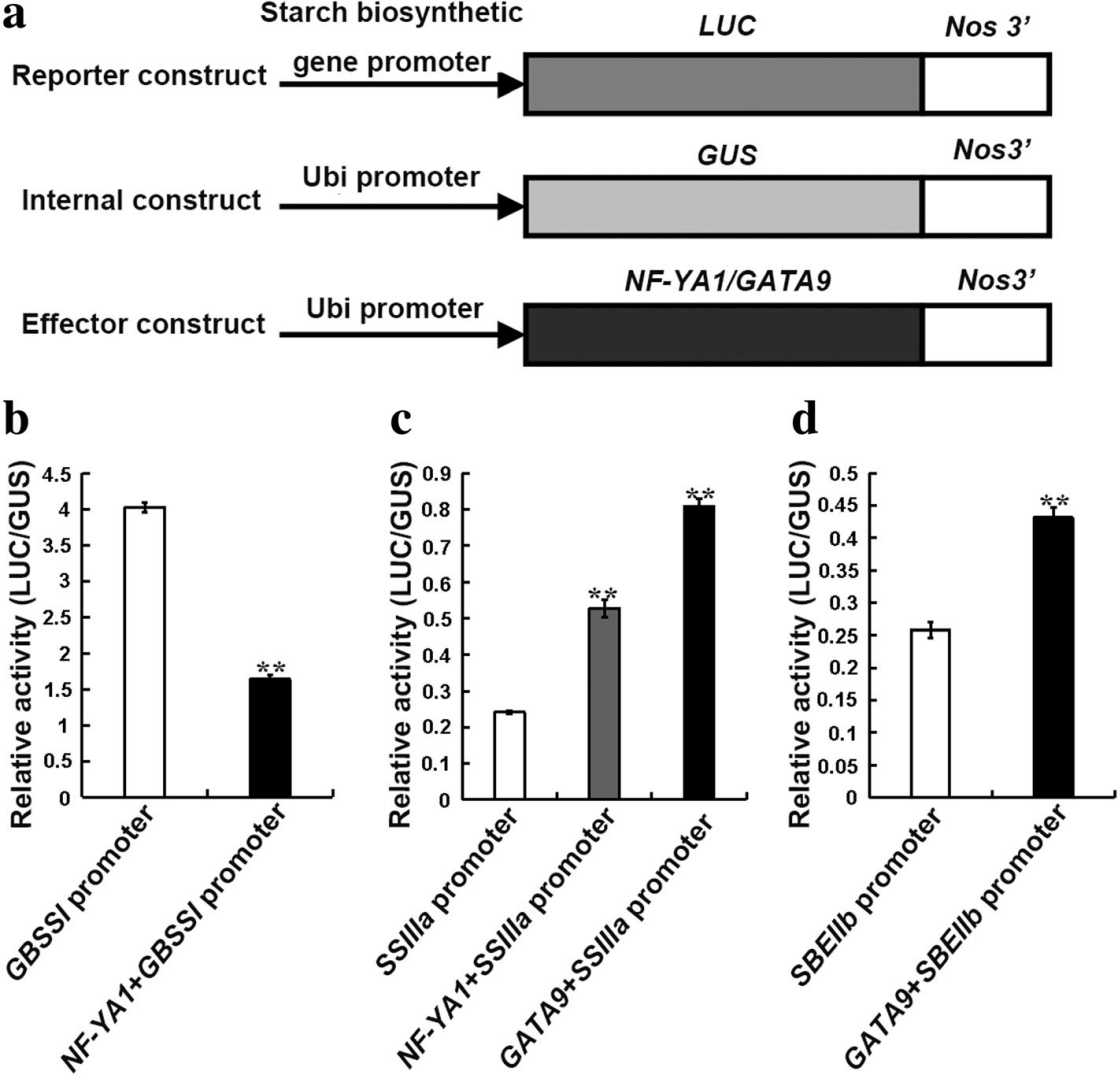
Transactivation assays of the promoters of *GBSSI*/*SSIIIa* and *SSIIIa*/*SBEIIb* by *NF-YA1* and *GATA9*, respectively. **a** Diagram of the effector, reporter, and internal constructs; **b** Regulatory interaction between *NF-YA1* and the promoter of *GBSSI*; **c** Regulatory interactions between *NF-YA1*/*GATA9* and the promoter of *SSIIIa*; **d** Regulatory interaction between *GATA9* and the promoter of *SBEIIb*. All data are means ± SD (*n* = 3). ** significant at *p* ≤ 0.01 by the Student’s *t* test

Furthermore, transient expression assays of miR169a, miR169o, *NF-YA1*, and *GATA9* in maize endosperm showed that *NF-YA1* was significantly down-regulated in miR169a-overexpressing endosperm, suggesting that miR169a could negatively regulate the expression of *NF-YA1*. *GATA9* was significantly up-regulated in miR169o-overexpressing endosperm, indicating that miR169o might positively regulate the expression of *GATA9* (Fig. 6). Meanwhile, *GBSSI* was significantly down-regulated, whereas *SSIIIa* was significantly up-regulated in *NF-YA1*-overexpressing endosperm; both *SSIIIa* and *SBEIIb* were significantly up-regulated in *GATA9*-overexpressing endosperm (Fig. 6). Moreover, transactivation assays of the promoters of *GBSSI*/*SSIIIa* and *SSIIIa/SBEIIb* by *NF-YA1* and *GATA9*, respectively, indicated that *NF-YA1* could enhance the promoter activity of *SSIIIa* and decrease the promoter activity of *GBSSI*; *GATA9* could enhance the promoter activities of *SSIIIa* and *GBSSI* (Fig. 7). These results demonstrated that miR169a-*NF-YA1* and miR169o-*GATA9* could be involved in the regulatory pathways for starch biosynthesis in maize endosperm. Overall, the regulatory interactions between miR169a-*NF-YA1*-*GBSSI*/*SSIIIa* and miR169o-*GATA9*-*SSIIIa*/*SBEIIb* that could affect target expression were confirmed in the present study.

## Discussion

### DEGs related to starch biosynthesis in developing maize endosperm

During endosperm development, active expression of most genes associated with primary metabolism can be observed [34, 35]. In the present study, transcriptome analysis of developing endosperm showed that most genes expressed differentially between Mo17 and Ji419 at 15 and 25 DAP participate in biological processes such as carbohydrate metabolism (Additional file 1: Figure S1a-d). As indicated in previous studies [26, 31, 36], these DEGs may directly or indirectly influence starch biosynthesis, resulting in divergent endosperm starch content and starch granule structure.

RNA-Seq data indicated that of the 33 differentially expressed starch biosynthetic genes, up-regulated genes outnumbered down-regulated genes in Ji419 (Fig. 1b), indicating that endosperm starch biosynthesis was more dynamic in Ji419 than in Mo17 at both 15 and 25 DAP, and possibly explaining why the endosperm starch content of Ji419 is higher than that of Mo17. In addition, some genes were up-regulated at one stage but down-regulated at the other. This might have contributed less to the difference in endosperm starch content between the two lines if the negative effect on endosperm starch content of the down-regulation of some genes at a specific stage had been offset by the up-regulation of other genes [36–38]. Here, *Sh2* was down-regulated, while *AGPL embryo* (*AGPLEMZM*) and *AGPb* were up-regulated in Ji419 at 25 DAP (Fig. 1a). Both *AGPLEMZM* and *AGPb*, which are homologous to *Sh2*, encode large subunits of AGP [37], and their up-regulation might counteract any negative effect of down-regulation of *Sh2*.

In the current study, transcript abundances of the eight key starch biosynthetic genes determined by qRT-PCR conformed to those determined from RNA-Seq data (Fig. 1c, d; Additional file 2: Table S1). Meanwhile, the expression patterns of these eight genes at 15, 20, 25, 30, 35, and 40 DAP (Fig. 2) were in accordance with results of previous research [39]. Among these genes, *Sh2* and *Bt2* determine the direct substrate for starch biosynthesis; *GBSSI* mainly plays a role in amylose biosynthesis, whereas *SSI*, *SSIIa*, *SSIIIa*, *SBEIIb*, and *ISA1* affect amylopectin biosynthesis [2, 5–7]. Mutations in these genes would result in reduced starch content or altered starch structure. For instance, the starch content of grains from *sh2* and *bt2* mutants is significantly lower than that from wild-type maize [40]. Mutations in *GBSSI* and *SBEIIb* cause alteration in the structure of starch [41, 42]. Therefore, differential expression of these starch biosynthetic genes might be at least one reason for the distinct endosperm starch content and starch granule structure of Mo17 and Ji419 (Additional file 3: Figure S2; Additional file 6: Figure S5). As regulation by miRNAs could be a major factor leading to the differential expression of these genes, the regulation by miRNAs of starch biosynthesis was further analyzed.

### miRNAs and corresponding targets associated with starch biosynthesis in developing endosperm

Although few miRNAs have been reported to regulate starch biosynthesis, miR156 can target SPL (SQUAMOSA PROMOTER-BINDING PROTEIN-LIKE)/SBP family transcription factors [43], and its overexpression in *Arabidopsis*, maize, and switchgrass results in significantly suppressed expression of SPL/SBP transcription factors and increased starch content [21]. SPL/SBP transcription factors can be involved in the initiation of lateral primordia, and can also influence the accumulation of carbohydrates [44, 45]. Thus, miR156-SPL/SBP transcription factors might participate in the regulation of starch biosynthesis. In the present study, two miR156 family members, miR156a-5p and miR156j-5p_R-1, might regulate starch biosynthesis by targeting GRMZM5G806833 (unknown function), GRMZM2G061734 (*SBP5*), and GRMZM2G156621 (*SBP31*) (Additional file 2: Tables S5 and S6). In rice, miR167 can regulate grain development through the auxin-miR167-ARF8-GH3.2 pathway [22]. miR393 can also target the auxin receptor genes *OsTIR1* and *OsAFB2* to modulate the development of roots and grains [46, 47]. Therefore, auxin-mediated miR167 and miR393 might participate in starch biosynthesis in endosperm. In the present study, miR167a-5p_R-2 might target GRMZM2G035405 (*ARF18*), GRMZM2G475882 (*ARF30*), and GRMZM2G078274 (*ARF6*), and miR393a-5p_L + 1R-2 could target GRMZM5G848945 (*AFB*) to modulate endosperm starch biosynthesis (Additional file 2: Tables S5 and S6). Growth regulators, a class of specific transcription factors in plants [48], play critical roles in the development of stems, leaves [49–51], roots [52–55], and grains [56, 57]. Notably, miR396 can modulate the expression of such growth regulators [48]. In rice, miR396c can regulate grain development and yield through the miR396c-GRF4 (growth regulating factor)-GIF1 (grain incomplete filling) pathway, which might be associated with starch biosynthesis [58]. In the present study, miR396a-5p, miR396c_L-1, and miR396e-5p_1ss21TA could target six growth regulator genes (GRMZM2G045977, GRMZM2G033612, GRMZM2G034876, GRMZM2G099862, GRMZM2G129147, and GRMZM2G041223) that might participate in the regulation of endosperm starch biosynthesis (Additional file 2: Tables S5 and S6).

Additionally, the newly identified miRNAs PC-5p-32802_53 and PC-5p-43820_42 were specifically expressed in the endosperm of Mo17 at 15 and 25 DAP, PC-3p-462671_5 and PC-5p-164091_13 were specifically expressed in Mo17 at 15 DAP, and PC-5p-97868_21 and PC-3p-24264_68 were specifically expressed in Mo17 at 25 DAP (Additional file 2: Tables S3 and S4). The target genes of these miRNAs, including GRMZM2G014793 (encoding a glycosyl transferase), GRMZM2G039906 (encoding a cell cycle control protein), and GRMZM2G473960 (encoding a seed maturation protein) (Additional file 2: Tables S5 and S6), may be closely associated with carbohydrate metabolism and grain development. Thus, these newly discovered miRNAs might regulate the differential expression of their target genes in the endosperm of Mo17 and Ji419. This could further differentially modulate biological processes such as starch biosynthesis and grain development, leading to divergent endosperm starch content and starch granule structure in these two lines (Additional file 3: Figure S2; Additional file 6: Figure S5).

### Regulatory network of starch biosynthesis in developing endosperm

Thus far, no research has been reported on an miRNA-mediated regulatory network for starch biosynthesis in maize. Our study of the co-expression of miRNA targets and known starch biosynthetic genes has established the existence of a complex miRNA-target regulatory network for starch biosynthesis in endosperm (Fig. 5). In this network, 19 miRNAs could target 19 genes to regulate endosperm starch biosynthesis, which reveals an miRNA-mediated mechanism modulating starch biosynthesis in developing maize endosperm.

In order to verify the regulatory network, gene expression patterns in four pathways involving miR169a-*NF-YA1*-*GBSSI*/*SSIIIa* and miR169o-*GATA9*-*SSIIIa*/*SBEIIb* were analyzed, and further transient expression and transactivation assays were conducted (Figs. 2, 6, and 7). The analyses of gene expression pattern in our study implied that miR169a and miR169o might negatively modulate their target genes *NF-YA1* and *GATA9*, respectively. The transient overexpression results indicated that miR169a could negatively regulate *NF-YA1*, whereas miR169o could positively regulate *GATA9* (Figs. 2 and 6). Most previous studies have shown that miRNAs can negatively regulate the expression of their target genes at the post-transcriptional level [59–63]. Also, there have been reports of positive regulation of target genes by miRNAs in humans and animals [64–66]. Therefore, it is possible that miR169o could positively regulate the target gene *GATA9*; however, further studies are needed to elucidate this mechanism. Nevertheless, it seemed that the regulatory relationship between miR169o and *GATA9* observed from expression pattern analysis was inconsistent with that from transient expression analysis. It might be because regulators other than miRNAs, such as transcription factors, hormones, or environmental signals could tune the expression of these target genes in normal maize, which would not be unexpected as the regulation of biological processes is often complex. In previous studies, NF-YA family transcription factors have been reported as the targets of miR169 [67–69]. Specifically, NF-YA1, NF-YA5, NF-YA6, and NF-YA9 play important roles in the development of male gametes, embryos, and seeds in *Arabidopsis* [70], and might be associated with starch biosynthesis. Two GATA family transcription factors, GNC and GNL/CGA1, can act as regulators of the gibberellin signaling pathway in *Arabidopsis* [71]. As gibberellin can regulate starch biosynthesis [18], GATA transcription factors might participate in starch biosynthesis through regulation of the gibberellin signaling pathway. In our study, the transient overexpression and transactivation results comprehensively confirmed that miR169a-*NF-YA1*-*GBSSI*/*SSIIIa* and miR169o-*GATA9*-*SSIIIa*/*SBEIIb* could interact and influence starch biosynthesis in maize endosperm (Figs. 6 and 7). In summary, the regulatory network described here will be of great significance for further in-depth research into the molecular mechanism of starch biosynthesis in developing maize endosperm. Moreover, although endosperm is the organ for synthesis and accumulation of starch, the evidence for maternal contribution to seed development (seed weight, seed size, etc.) in maize has revealed that starch content may be also controlled by maternal effect [72, 73]. Therefore, a comprehensive study on the coordinated roles of maternal effect and starch biosynthetic pathways will be further considered for a better understanding of the mechanism of starch biosynthesis in seed, which will facilitate the improvement of grain yield and quality in future maize breeding programs.

## Methods

### Plant materials

Two maize inbred lines, Mo17 and its improved line Ji419, were grown at the Changping Station at the Institute of Crop Science, Chinese Academy of Agricultural Sciences in 2015. Ji419 was derived from a cross between B68^Ht^ and Mo17. These two lines differ significantly in endosperm starch content and starch granule structure (Additional file 3: Figure S2; Additional file 6: Figure S5). Further details of the kernel morphological characteristics of Mo17 and Ji419 have been detailed in our previous study [74]. The experimental field consisted of 40 rows that were 3 m long and 0.6 m wide, with spacing of 0.3 m between plants within rows. Common cultural practices for growing maize were used. Endosperm tissues were collected from Mo17 and Ji419 at 15, 20, 25, 30, 35, and 40 DAP. Three biological replicates were sampled, with each replicate sampled from a separate ear chosen from each line. The endosperm samples from kernels sampled from the central portion of the ear were frozen immediately in liquid nitrogen and stored at − 80 °C.

### RNA extraction, library construction, and sequencing

Total RNA was extracted from each sample using Trizol reagent (Invitrogen, Carlsbad, USA). The quantity and purity of the total RNA were checked using NanoDrop ND-1000 (NanoDrop, Wilmington, USA) and Agilent 2100 with a minimum RNA Integrity Number (RIN) threshold value > 7.0. Approximately 15 μg of total RNA were used to prepare each transcriptome sequencing library, and a total of 12 libraries were constructed for each biological replicate of the Mo17 and Ji419 endosperm samples taken at 15 and 25 DAP. RNA-Seq was performed by OE-Biotech Co., Ltd., Shanghai, China.

Approximately 1 μg of total RNA was used to prepare a small RNA library according to the protocol of TruSeq Small RNA Sample Prep Kit (Illumina, San Diego, USA), and four libraries were constructed from equally mixed RNA quantities from the three biological replicates of the Mo17 and Ji419 endosperm samples taken at 15 and 25 DAP. Approximately 20 μg of total RNA were used to prepare a degradome library, and two libraries were constructed from equally mixed RNAs from the six endosperm samples of Mo17 and Ji419 at both 15 and 25 DAP. Both small RNA and degradome sequencing were performed on an Illumina HiSeq 2500 platform at the LC-Biotech Co., Ltd., Hangzhou, China, following the manufacturer’s recommended protocol.

### Transcriptome analysis

Clean reads were obtained after filtering adaptor sequences, and empty, low-quality, and one-copy tags from the raw reads. Next, reads were aligned to the maize reference gene set based on the B73 genome (release 5b.60) using the SOAPaligner/SOAP2 program [75]. Gene expression was quantified as fragments per kb per million reads (FPKM) [76]. Genes with *P*-value less than 0.05 were considered to have altered expression and were designated as DEGs.

DEGs were subjected to GO functional enrichment and KEGG pathway analyses. GO annotation was conducted using Blast2GO software (v.2.5.0) [77], and the significance of enrichment of each functional term was computed using the hypergeometric distribution test. KEGG Automatic Annotation Server (KAAS) provides functional annotation of genes by blast against the manually managed KEGG database. The results contain KEGG Orthology (KO) assignments and automatically generated KEGG pathways.

Forty known starch biosynthetic genes [78] were chosen to construct pathways for starch biosynthesis, and the expression profiles of these genes were also determined from the RNA-Seq data. Further, the expression of eight key starch biosynthetic genes (*GBSSI*, *SSI*, *SSIIa*, *SSIIIa*, *ISA1*, *SBEIIb*, *Sh2*, and *Bt2*) in Mo17 and Ji419 at 15 and 25 DAP was validated using qRT-PCR. The first-strand cDNAs of these eight genes were synthesized using the Fast Quant RT Kit (TianGen, Beijing, China). Then qRT-PCR was carried out using the Bio-Rad iQ5 (Bio-Rad, USA) following the instructions for the SuperReal PreMix Plus (SYBR Green) (TianGen, Beijing, China). Three technical replicates of all reactions were performed, and 18S rRNA was used as the internal reference for expression. The primers used for qRT-PCR were listed in Additional file 2: Table S9.

### miRNA analysis

Raw reads were initially processed by an in-house program, ACGT101-miR (LC Sciences, Houston, USA). Clean reads were obtained after filtering adapters, low-quality reads, common RNA families (rRNA, tRNA, snRNA, snoRNA), and repeats. The filtered unique sequences with length in 18 to 25 nucleotides were mapped to the precursors of specific species in miRBase 20.0 by blast to identify known miRNAs and previously unknown 5p- and 3p-derived miRNAs. Length variation at both 3′ and 5′ ends and one mismatch inside the sequences were allowed in the alignment. Unique sequences that match one of the arms in the stem-loops of mature miRNAs of specific species were identified as known miRNAs. Any unique sequences that mapped to the other arm of the stem-loops of precursors of known specific species opposite to the annotated mature miRNA-containing arm were considered to be candidates for previously unknown 5p- or 3p-derived miRNAs. The remaining sequences were mapped to the precursors of other selected species (with the exclusion of specific species) in miRBase 20.0 by blast, and the mapped pre-miRNAs were used as further blast queries against the specific species genomes to determine their genomic locations. The miRNAs in the above two categories were then defined as known miRNAs. The unmapped sequences were aligned against the specific genomes, and the hairpin RNA structures containing sequences were predicated from the flanking 120 nucleotides sequences using RNAfold software. miRNAs with at least twofold change in expression (Log_2_FC ≥ 1) and at least 10 reads in a dataset were identified here as differentially expressed miRNAs.

### Degradome analysis

Potentially cleaved targets of 20 to 21 nucleotides in the extracted sequence reads generated by degradome sequencing were identified using CleaveLand 3.0. The degradome reads were then mapped to the transcriptome data. We constructed t-plots using the transcriptome data for highly efficient analysis of potential miRNA targets. Finally, the targets of differentially expressed miRNAs were identified as candidate target genes.

### Co-expression analysis of candidate targets and eight starch biosynthetic genes

Using our RNA-Seq data, as well as those from the study performed by Li et al. [35], co-expression analysis among the candidate target genes and eight key starch biosynthetic genes (*Sh2*, *Bt2*, *GBSSI*, *SSI*, *SSIIa*, *SSIIIa*, *ISA1*, and *SBEIIb*) was conducted in SPSS software (v.24.0). Candidate target genes were considered associated with endosperm starch biosynthesis only for Pearson’s correlation coefficient of ≥0.60 between their expression and that of starch biosynthetic gene(s). Based on the co-expression analysis, a model molecular regulatory network of miRNA-target-starch biosynthesis in developing maize endosperm was constructed using Cytoscape software (v.3.6.0).

### Expression pattern analysis

The expression patterns of the pathways involving miR169a-*NF-YA1*-*GBSSI*/*SSIIIa* and miR169o-*GATA9*-*SSIIIa*/*SBEIIb* were analyzed by qRT-PCR. Total RNAs and miRNAs from Mo17 and Ji419 at 15, 20, 25, 30, 35, and 40 DAP were used for analysis of gene expression patterns by qRT-PCR to clarify any possible regulatory relationship between the chosen miRNA-target pairs in developing maize endosperm. Two target genes, *NF-YA1* and *GATA9*, were analyzed using qRT-PCR as described above. The first-strand cDNAs of these miRNAs were synthesized using the miRcute miRNA First-Strand cDNA Synthesis Kit (TianGen, Beijing, China). qRT-PCR was then carried out to analyze expression of these miRNAs using the Bio-Rad iQ5 (Bio-Rad, USA) following the instructions for the miRcute miRNA qRT-PCR Detection Kit (SYBR Green) (TianGen, Beijing, China). Three technical replicates were also performed, and 5S rRNA was used as internal reference. The primers used for qRT-PCR were listed in Additional file 2: Table S9.

### Transient expression analysis

The pre-miR169a/169o precursor and the coding sequences of *NF-YA1* and *GATA9* were cloned and inserted into PUbi:Gus by replacing the β-glucuronidase (Gus) reporter gene and driven by the maize ubiquitin promoter (pUbi:miR169a, pUbi:miR169o, pUbi:*NF-YA1* and pUbi:*GATA9*). Transient overexpression of the above vectors in maize endosperm was mediated by particle bombardment, and the pUbi:Gus was used as a control. Three biological replicates were performed. The endosperm used for particle bombardment was isolated from immature maize kernels (10 DAP), which had been surface-sterilized with 75% ethanol and plated on MS medium (Murashige and Skoog salts containing 0.85% agar and 12% sucrose) for 4 h prior to bombardment. Particle bombardment was performed as previously described [16]. After particle bombardment, the medium plates containing bombarded endosperm were incubated at 28 °C in the dark for 16–24 h. Total RNA was extracted from the endosperm, and the expression of related miRNAs, target genes, and starch biosynthetic genes were analyzed using qRT-PCR as described above to characterize the starch biosynthetic pathways. The primers used for qRT-PCR were listed in Additional file 2: Table S9.

### Transactivation assay

To determine the regulatory relationship between *NF-YA1* and the promoters of *GBSSI*/*SSIIIa*, and that between *GATA9* and the promoters of *SSIIIa*/*SBEIIb*, transactivation assays were conducted. pUbi:*NF-YA1* and pUbi:*GATA9* served as effector constructs. The promoters of *GBSSI*, *SSIIIa*, and *SBEIIb* were cloned into the reporter construct pPromoter:Luc (p*GBSSI*:Luc, p*SSIIIa*:Luc, and p*SBEIIb*:Luc). The pUbi:Gus was used as an internal construct. The effector construct, the reporter construct, and the internal construct were combined at a molar ratio of 2:2:1 and then co-bombarded into maize endosperm. Three biological replicates were performed. After the medium plates containing bombarded endosperm were incubated at 28 °C in the dark for 24 h, the activity of Gus and Luc were analyzed using a Luminoskan™ Ascent (Thermo, USA). The primers used for transactivation assays were listed in Additional file 2: Table S9.

## Additional files

Additional file 1: **Figure S1.** Analysis of GO term and KEGG pathway enrichment among genes differentially expressed in the endosperm of maize inbred lines Mo17 and Ji419 at 15 and 25 DAP. **a-b** GO terms of differentially expressed genes in the endosperm of Mo17 and Ji419 at 15 DAP (**a**) and 25 DAP (**b**); **c-d** KEGG pathways of differentially expressed genes in the endosperm of Mo17 and Ji419 at 15 DAP (**c**) and 25 DAP(**d**). (TIF 1660 kb)

Additional file 2: **Table S1.** The expression of 40 starch biosynthetic genes in the endosperm of maize inbred lines Mo17 and Ji419 at 15 and 25 DAP; **Table S2.** GO term and KEGG pathway enrichment of 40 starch biosynthetic genes in the endosperm of maize inbred lines Mo17 and Ji419 at 15 and 25 DAP; **Table S3.** Differentially expressed miRNAs in the endosperm of maize inbred lines Mo17 and Ji419 at 15 DAP; **Table S4.** Differentially expressed miRNAs in the endosperm of maize inbred lines Mo17 and Ji419 at 25 DAP; **Table S5.** Differentially expressed miRNAs and their target genes in the endosperm of maize inbred lines Mo17 and Ji419 at 15 DAP; **Table S6.** Differentially expressed miRNAs and their target genes in the endosperm of maize inbred lines Mo17 and Ji419 at 25 DAP; **Table S7.** Predicted target genes of miRNAs in the endosperm of maize inbred lines Mo17 and Ji419 at 15 and 25 DAP; **Table S8.** Pearson’s correlation coefficients between the expression of 35 target genes and eight key starch biosynthetic genes in the endosperm of maize inbred lines Mo17 and Ji419 at 15 and 25 DAP; **Table S9.** Primers used in this study. (XLSX 508 kb)

Additional file 3: **Figure S2.** Starch content in the mature endosperm of maize inbred lines Mo17 and Ji419. All data are means ± SD (*n* = 3). *, ** significant at *p* ≤ 0.05 and *p* ≤ 0.01 by the student’s *t* test. (TIF 840 kb)

Additional file 4: **Figure S3.** Size distribution of unique miRNAs (**a**) and Venn diagram of miRNAs differentially expressed (**b**) in the endosperm of maize inbred lines Mo17 and Ji419 at 15 and 25 DAP. Red represents miRNAs down-regulated in Ji419 compared with Mo17 at 15 DAP; blue represents miRNAs up-regulated in Ji419 at 15 DAP; yellow represents miRNAs down-regulated in Ji419 at 25 DAP; green represents miRNAs up-regulated in Ji419 at 25 DAP. (TIF 358 kb)

Additional file 5: **Figure S4.** Heat map depicting co-expression analysis based on Pearson’s correlation coefficients between the expression of 35 target genes and eight key starch biosynthetic genes in the endosperm of maize inbred lines Mo17 and Ji419 at 15 and 25 DAP. (TIF 401 kb)

Additional file 6: **Figure S5.** Scanning electron micrograph of starchy endosperm cells in seeds of maize inbred lines Mo17 and Ji419 at different developmental stages. **a** Endosperm of Mo17 at 15 DAP; **b** Endosperm of Mo17 at 25 DAP; **c** Endosperm of Mo17 at 35 DAP; **d** Mature endosperm of Mo17; **e** Endosperm of Ji419 at 15 DAP; **f** Endosperm of Ji419 at 25 DAP; **g** Endosperm of Ji419 at 35 DAP; **h** Mature endosperm of Ji419. Scale bar, 50 μm. (TIF 3589 kb)

## Data Availability

Raw sequence data for small RNAs, degradome, and transcriptome in this study are available at the NCBI Sequence Read Archive (http://www.ncbi.nlm.nih.gov/Traces/sra) under accession number SRP099290.

## Abbreviations

ABA: Abscisic acid
AFB: Auxin signaling F-box
AGP: ADP-glucose pyrophosphorylase
AGPL: AGP large subunit
AGPLEMZM: AGPL embryo
AGPS: AGP small subunit
ARF: Auxin response factor
BR: Brassinosteroid
Bt2: Brittle2
CA2P3: CCAAT-HAP2-transcription factor 23
CA2P4: CCAAT-HAP2-transcription factor 24
COPB1: Coatomer protein complex subunit beta 1
DAP: D after pollination
DBE: Starch debranching enzyme
DEGs: Differentially expressed genes
FPKM: Fragments per kb per million reads
GA: Gibberellin
GATA9: C2C2-GATA-transcription factor 9
GBSS: Granule-bound starch synthase
GIF: Grain incomplete filling
GO: Gene Ontology
GRAS: GIBBERELLIN-ACID INSENSITIVE (GAI), REPRESSOR OF GAI (RGA), SCARECROW (SCR)
GRF: Growth regulating factor
Gus: β-glucuronidase
IAA: Auxin
ISA1: Isoamylase 1
KAAS: KEGG Automatic Annotation Server
KEGG: Kyoto Encyclopedia of Genes and Genomes
KO: KEGG Orthology
miRNAs: microRNAs
NF-YA1: Nuclear transcription factor Y subunit A1
O2: opaque2
PBF: Prolamine-box binding factor
PHI1: Phosphohexose isomerase 1
PHO: Starch phosphorylase
PPDK1: Pyruvate orthophosphate dikinase 1
qRT-PCR: quantitative real-time polymerase chain reaction
RIN: RNA Integrity Number
RNA-Seq: RNA sequencing
RSR1: Rice Starch Regulator 1
SBE: Starch branching enzyme
SBP5: Squamosa promoter-binding protein transcription factor 5
Sh2: Shrunken2
SPL: Squamosa Promoter-binding Protein-Like
SS: Soluble starch synthase
SUSIBA: Sugar signaling in barley
UGP: UDP-glucose pyrophosphorylase
