# Supplementary figures and images for: Integrated transcriptome, small RNA, and degradome analysis reveals the complex network regulating starch biosynthesis in maize
Source: BMC Genomics. 2019 Jul 11;20:574. doi: 10.1186/s12864-019-5945-1 (PMC6625009; doi:10.1186/s12864-019-5945-1)

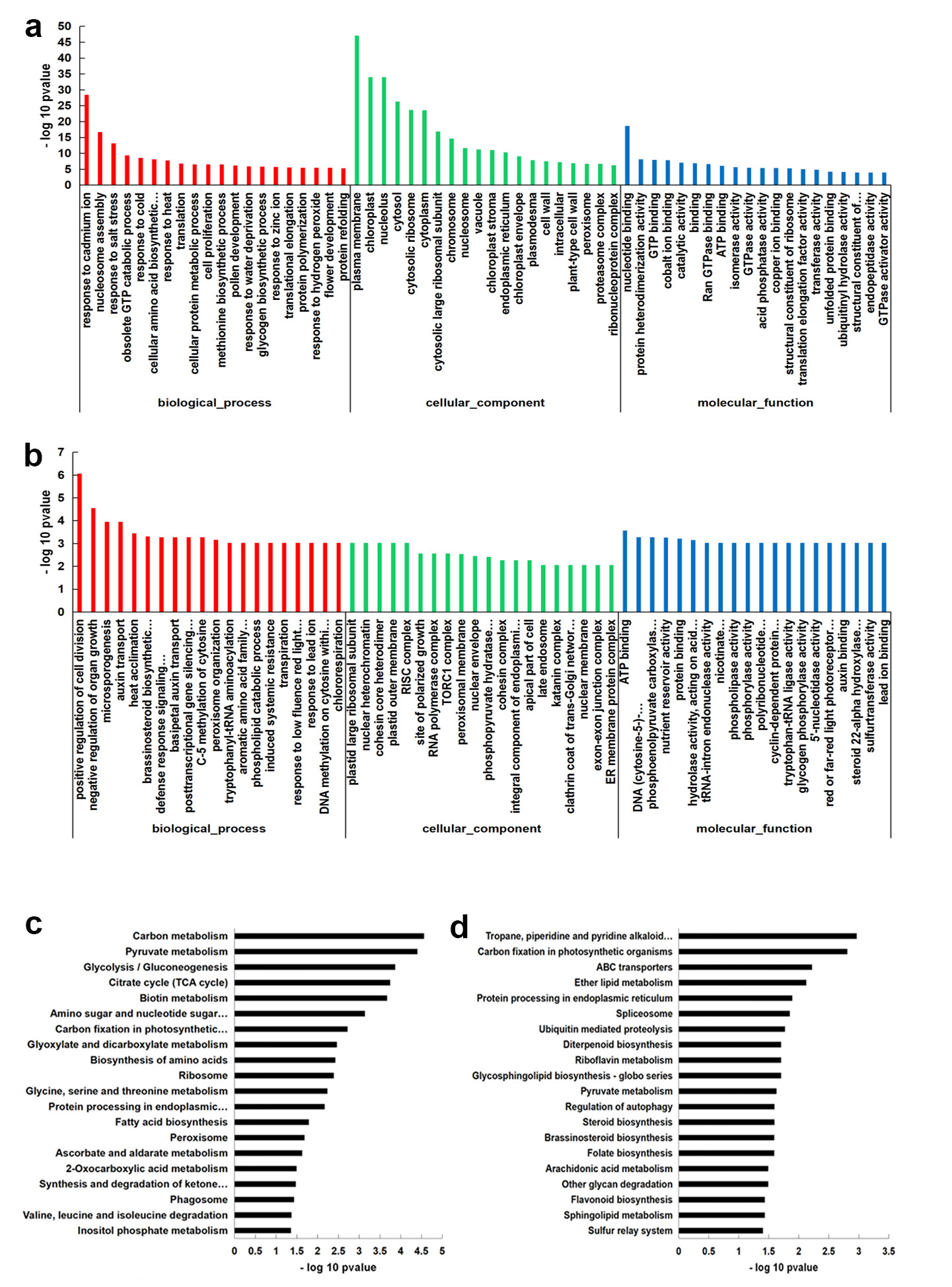

Supplement: Supplementary file 1 — Figure S1. Analysis of GO term and KEGG pathway enrichment among genes differentially expressed in the endosperm of maize inbred lines Mo17 and Ji419 at 15 and 25 DAP. a-b GO terms of differentially expressed genes in the endosperm of Mo17 and Ji419 at 15 DAP (a) and 25 DAP (b); c-d KEGG pathways of differentially expressed genes in the endosperm of Mo17 and Ji419 at 15 DAP (c) and 25 DAP(d). (TIF 1660 kb) [file 12864_2019_5945_MOESM1_ESM.tif]

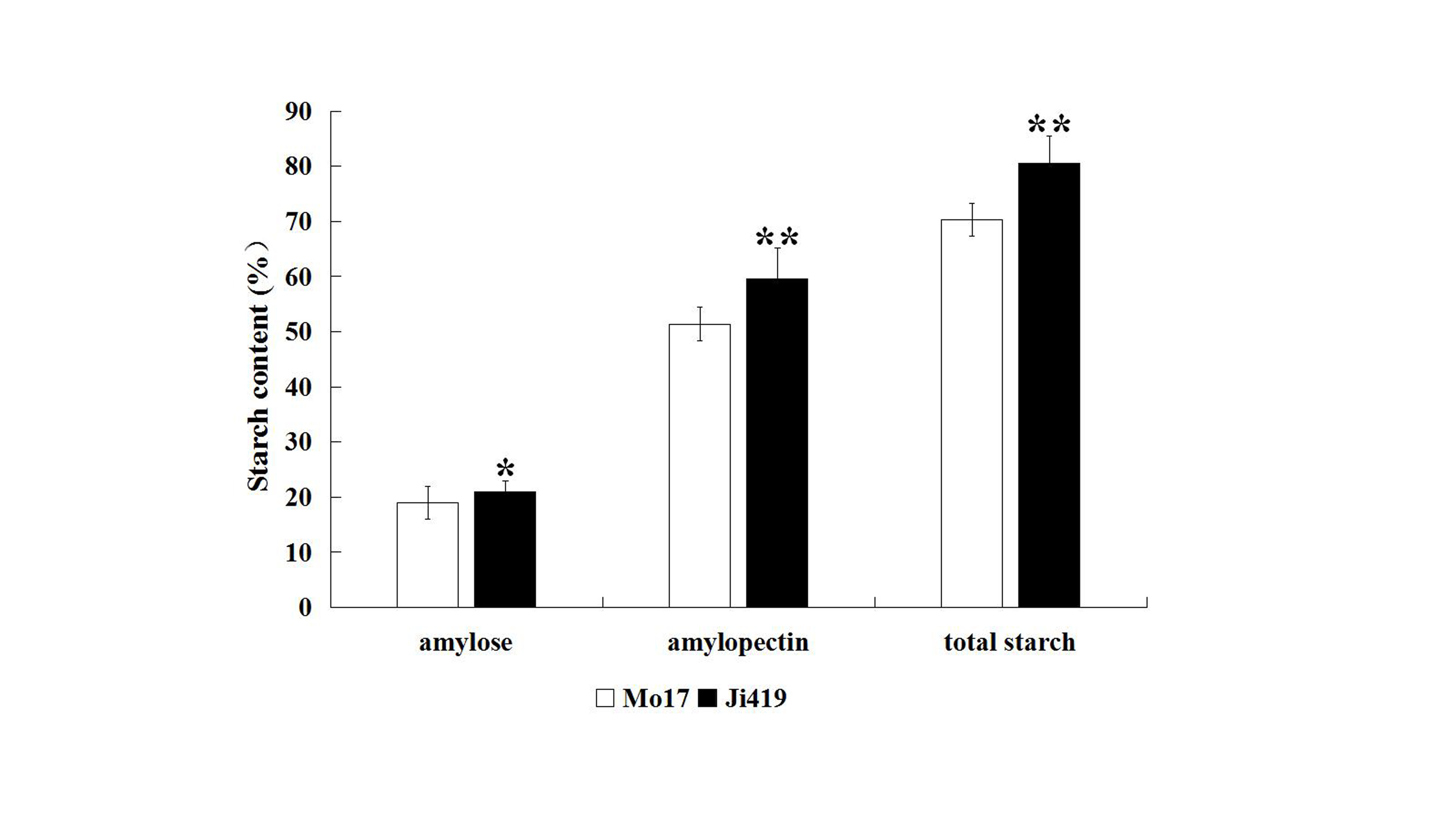

Supplement: Supplementary file 3 — Figure S2. Starch content in the mature endosperm of maize inbred lines Mo17 and Ji419. All data are means ± SD (n = 3). *, ** significant at p ≤ 0.05 and p ≤ 0.01 by the student’s t test. (TIF 840 kb) [file 12864_2019_5945_MOESM3_ESM.tif]

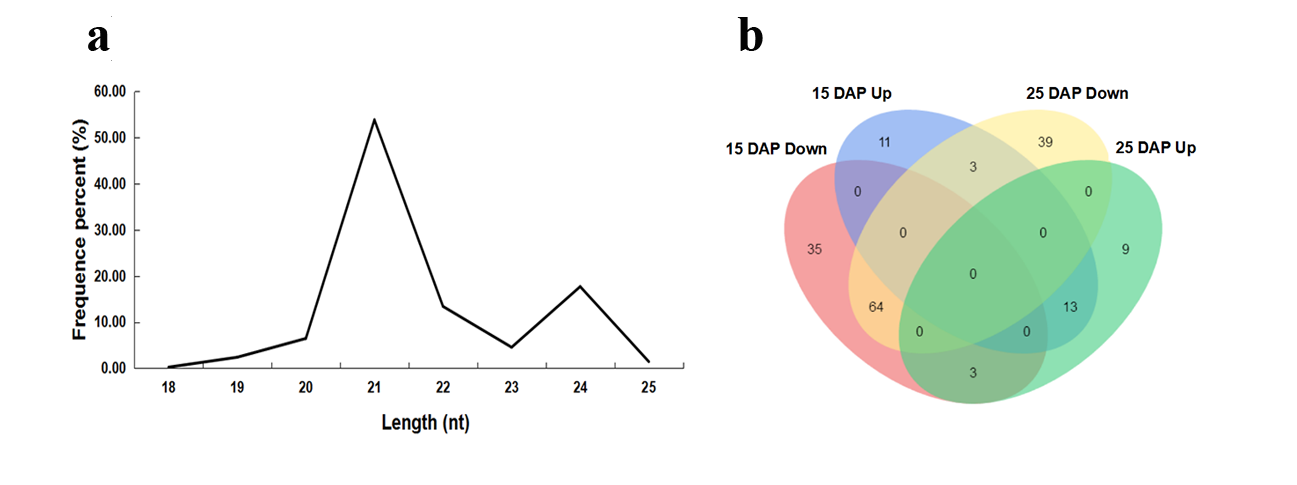

Supplement: Supplementary file 4 — Figure S3. Size distribution of unique miRNAs (a) and Venn diagram of miRNAs differentially expressed (b) in the endosperm of maize inbred lines Mo17 and Ji419 at 15 and 25 DAP. Red represents miRNAs down-regulated in Ji419 compared with Mo17 at 15 DAP; blue represents miRNAs up-regulated in Ji419 at 15 DAP; yellow represents miRNAs down-regulated in Ji419 at 25 DAP; green represents miRNAs up-regulated in Ji419 at 25 DAP. (TIF 358 kb) [file 12864_2019_5945_MOESM4_ESM.tif]

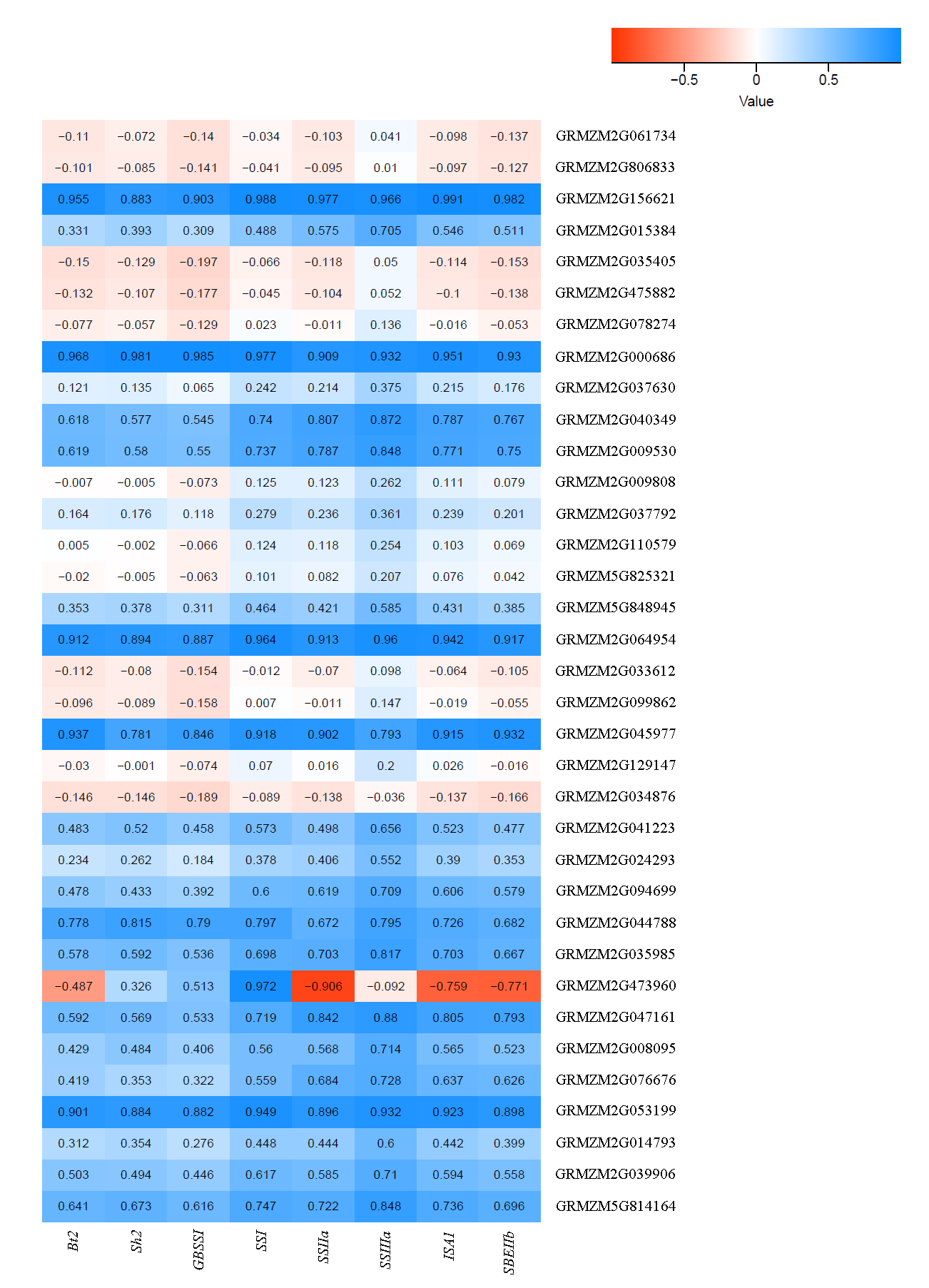

Supplement: Supplementary file 5 — Figure S4. Heat map depicting co-expression analysis based on Pearson’s correlation coefficients between the expression of 35 target genes and eight key starch biosynthetic genes in the endosperm of maize inbred lines Mo17 and Ji419 at 15 and 25 DAP. (TIF 401 kb) [file 12864_2019_5945_MOESM5_ESM.tif]

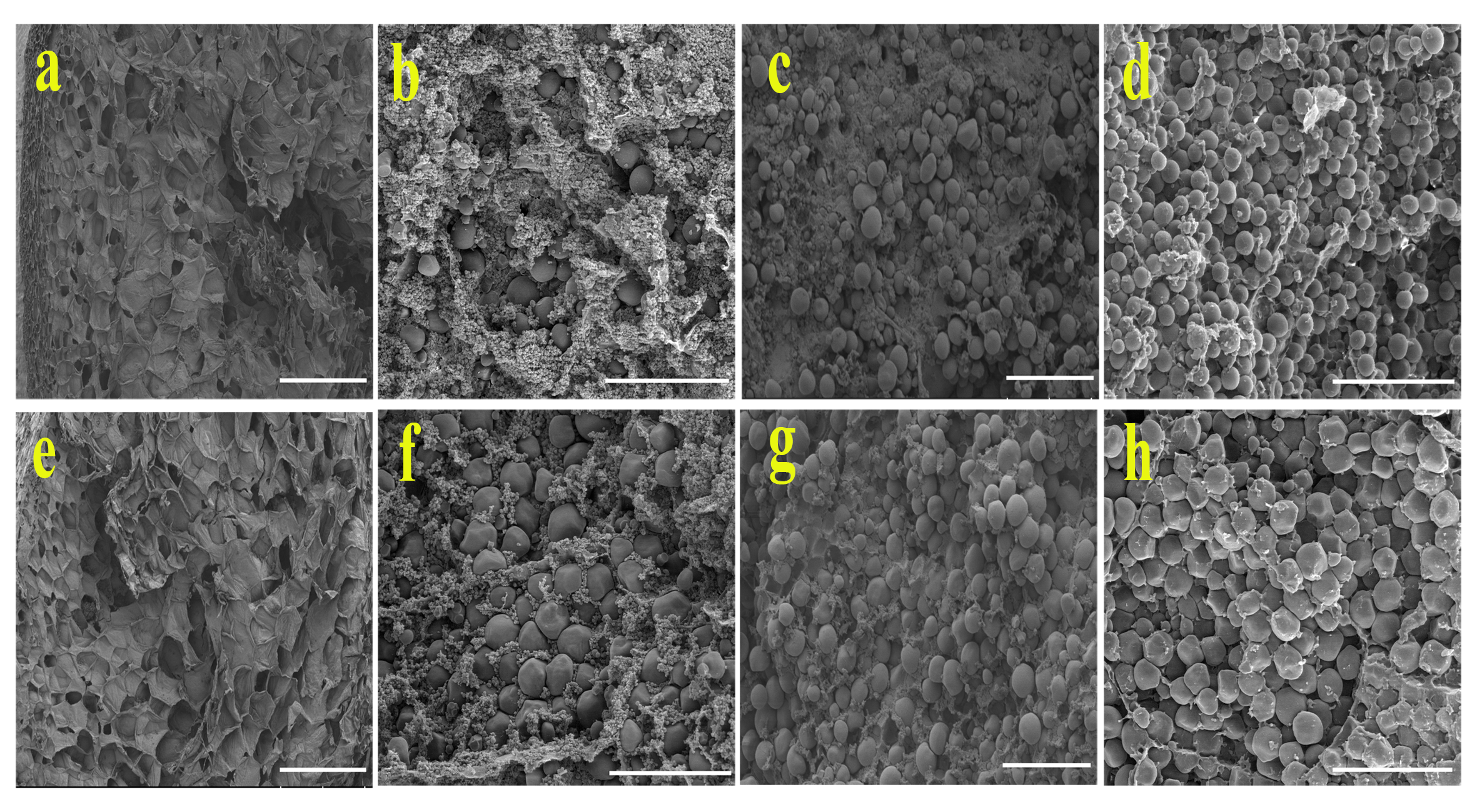

Supplement: Supplementary file 6 — Figure S5. Scanning electron micrograph of starchy endosperm cells in seeds of maize inbred lines Mo17 and Ji419 at different developmental stages. a Endosperm of Mo17 at 15 DAP; b Endosperm of Mo17 at 25 DAP; c Endosperm of Mo17 at 35 DAP; d Mature endosperm of Mo17; e Endosperm of Ji419 at 15 DAP; f Endosperm of Ji419 at 25 DAP; g Endosperm of Ji419 at 35 DAP; h Mature endosperm of Ji419. Scale bar, 50 μm. (TIF 3589 kb) [file 12864_2019_5945_MOESM6_ESM.tif]
